# High-Polarization-Discriminating Infrared Detection Using a Single Quantum Well Sandwiched in Plasmonic Micro-Cavity

**DOI:** 10.1038/srep06332

**Published:** 2014-09-11

**Authors:** Qian Li, ZhiFeng Li, Ning Li, XiaoShuang Chen, PingPing Chen, XueChu Shen, Wei Lu

**Affiliations:** 1 National Laboratory for Infrared Physics, Shanghai Institute of Technical Physics, Chinese Academy of Sciences, 500 Yutian Road, Shanghai 200083, P. R. China and Synergetic Innovation Center of Quantum Information & Quantum Physics, University of Science and Technology of China, Hefei, Anhui 230026, P. R. China

## Abstract

Polarimetric imaging has proved its value in medical diagnostics, bionics, remote sensing, astronomy, and in many other wide fields. Pixel-level solid monolithically integrated polarimetric imaging photo-detectors are the trend for infrared polarimetric imaging devices. For better polarimetric imaging performance the high polarization discriminating detectors are very much critical. Here we demonstrate the high infrared light polarization resolving capabilities of a quantum well (QW) detector in hybrid structure of single QW and plasmonic micro-cavity that uses QW as an active structure in the near field regime of plasmonic effect enhanced cavity, in which the photoelectric conversion in such a plasmonic micro-cavity has been realized. The detector's extinction ratio reaches 65 at the wavelength of 14.7 μm, about 6 times enhanced in such a type of pixel-level polarization long wave infrared photodetectors. The enhancement mechanism is attributed to artificial plasmonic modulation on optical propagation and distribution in the plasmonic micro-cavities.

Light polarization provides richer sets of descriptive physical constraints for the interpretation of the imaged scene in addition to intensity and wavelength^1^. Polarimetric imaging has been intensively developed in various fields of applications. In modern medicine it could become a fast and accurate optical method for detecting cancer and determining the stage of the disease^2^. In bionics it helps making machine vision to detect motion/collision of moving objects^1^. In military reconnaissance it is used to single out man-made objects from camouflages when there is little contrast in intensity imagery^3^. In remote sensing it can even monitor the change of the earth because human activities often change the polarization state of the electromagnetic (EM) energy reflected or emitted from the surface of the earth^4^. Conventional polarization measurements employ time-sequenced or simultaneous measurements by using various stand-alone polarizers and retarders to obtain the polarization Stokes parameters^5^. Free standing polarizers' extinction ratio can easily reach 100 or even higher, satisfying the most requirements of static and indoor measurements. For fast moving objects or field measurements, recent developments involve micro-polarizers that are directly integrated into the structure of individual detecting pixels to form polarization detecting focal plane arrays (FPAs) so that real-time fast polarimetric imaging are very much facilitated to sustain the applications in, for example, air- or satellite-born remote sensing. The extinction ratio of micro-polarizers, however, is far less than their stand-alone counterparts. People have been seeking high extinction ratio wire-grid micro-polarizers to acquire more sensitive all-solid polarimetric imagers. Once succeeded, especially in long wave infrared (LWIR) with the wavelength longer than about 8 μm where the polarization signatures are dominated by emission from target and can be very stable in time when scene temperatures are stable^3^, application opportunities will be widely open, for example, like live-tumor diagnosis and any other meticulous target recognitions. In visible range of the spectrum the extinction ratio of micro-polarizers has reached ~60^6^. In infrared (IR) range, however, the extinction ratio is only 2.3 for IR detectors^7^ (expressed as contrast ratio (diattenuation) = (Rx-Ry)/(Rx+Ry) > 40% in the referenced paper), and will saturate to only about 10 (responsivity contrast ~82%) even when the pixel size is enlarged to 100 μm^8^. The reason might be attributed to the cross-talks between neighbor pixels or the wave-leakage through pixel edges that contaminates the polarization as for imaging FPAs the detecting pixel is completely exposed within the light beam and the longer wavelength in IR range will make the cross-talk/wave-leakage much easier.

Recent intensively studied plasmonics^9^,^10^,^11^ has demonstrated strong capabilities on manipulating light-matter coupling by modifying the incident optical field and forming resonant condition for photoelectric conversion in near- and far-field regimes. Micro-cavities have displayed an excellent avenue to greatly enhance the field intensity by eliminating far-field effects, which has made a direct observation of individual quantum particles^12^, leading to a very high intense nanolaser beams^13^, etc. In this article we report the achievement on the combination of plasmonics and micro-cavities with the insertion of an active layer for photo-detections. By manipulating the localized surface plasmon (LSP) mode and the surface plasmon polariton (SPP) mode we have fulfilled the modulations of wave propagation and field distribution of IR photons in all-solid wire-grid polarizer scheme, realizing high extinction ratio of 65 at LWIR of 14.7 μm.

## Results

### Device structure of the PCQWID

**Figure 1:**
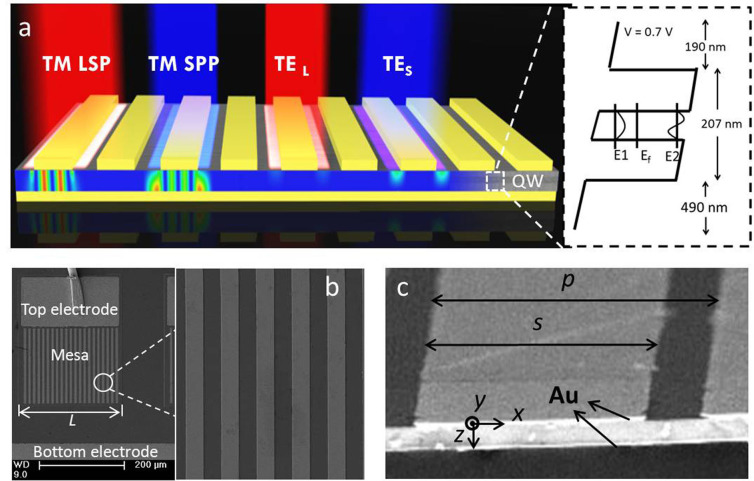
Schematic diagraph of the PCQWID. (a) The incident TM and TE polarized lights have different coupling behaviors to form different cavity modes. The figure shows the electric field distributions of TM LSP (Localized Surface Plasmon) mode at its calculated resonant wavelength of 14.9 μm, TM SPP (Surface Plasmon Polariton) mode at its calculated resonant wavelength of 12.4 μm, and TE modes at the same wavelength of 14.9 and 12.4 μm, respectively. The right panel is the schematics of the active layer. The first two energy levels of the single QW are separated of 97 meV, corresponding to a wavelength of 12.7 μm. (b) SEM image of the cleaved facet of the cavity structure. Geometrical parameters are designed as *p* = 9.2~10.6 μm, *s* = 4.9~5.9 μm. Both the top and bottom Au layers are 100 nm thick. (c) SEM image of a fabricated PCQWID, the grating covering area is 230 × 200 μm^2^.

The configuration and operation principles of our grating Plasmonic micro-Cavity Quantum Well Infrared Detector (PCQWID) are shown in Figure 1, together with the SEM images of the fabricated PCQWID and the cleaved facet. A single GaAs/Al_0.13_Ga_0.87_As QW with the thickness of 207 nm and the two contact layers of 190 nm and 490 nm are sandwiched between the upper Au periodic grating and the bottom Au reflection layer whose thicknesses are both 100 nm. The grating covering area is 230 × 200 μm^2^. The top and bottom electrodes as well as the mesa-isolation boundary are shown in Figure 1 b. Sample preparation processes can be found in Supporting Information (SI) S1. The small distance, about 900 nm, between the two Au layers makes them couple with each other and form a plasmonic micro-cavity, squeezing the incident light into the cavity. When a non-polarized light illuminates, the transverse magnetic (TM) and transverse electric (TE) polarized light will have different coupling properties. At the wavelength of plasmonic resonance for TM light, most of TM light will couple into the cavity. The light wavevector will be transformed from *z* to *x* direction, generating strong electrical components along *z* direction, *E_z_*, which can be absorbed by QW with inter-subband transition processes^14^. TE light will have different resonant wavelength and will be weakly coupled. It will have the electrical components in *y* direction, which is forbidden for the inter-subband transition by the quantum selection rule^14^. The inter-subband transition in QW induces photocurrents in the biased PCQWID, with which we obtain high contrast responses to TM and TE light.

### Polarization discriminating ability of PCQWID

In ideal cases the TM light can generate photocurrent while the TE light is forbidden, the non-ideal factors, however, in the structural and fabrications of the PCQWID will introduce scatterings, creating *E_z_* for TE light and reducing *E_z_* for TM light. They are non-intrinsic photocurrents in PCQWID, which will contaminate the polarization. To quantitatively analyze the polarization discriminating capability, we introduce Equation 1 to express the extinction ratio at the cavity resonant wavelength in our hybrid structured PCQWID, defined as 
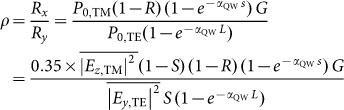
where *ρ* is the polarization extinction ratio, *R_x_* and *R_y_* are the detector responses to the lights polarized along *x* (TM) and *y* (TE) directions, respectively. *P*_0,TM_ and *P*_0,TE_ are the effective optical power in TM and TE light within the cavity, respectively. *R* is the lateral reflectivity at the interfaces of Fabry-Perot resonators formed between the single-metal and double-metal regions^15^. *α*_QW_ is the QW absorption coefficient. *s* and *L* are the widths of metal strip and detector mesa, respectively. *G* and *S* are enhancement and scattering factors that will be described in the later text. 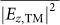 and 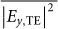 are the simulated average relative intensities of electric field by taking the input electric field intensity in the incident optical beam as the unity. 0.35 is the transmissivity of the polarizer used in the experiment. The detailed explanation and calculations of Equation 1 are described in SI S5. According to the selection rule, only *E_z_* component contributes to the photo-electric conversion. So *P*_0,TM_ and *P*_0,TE_ are the effective *E_z_* in TM and TE light, respectively. In TE light originaly there is no *E_z_* component. *P*_0,TE_ is induced by the *E_z_* scattered from *E_y_*. We introduce a scattering factor *S* whose value represents the ratio of *E_z_* component scattering into *E_y_* component for TM light (TM loss) and *E_y_*scattering into *E_z_* component for TE light (TE leakage), which are resulted from the extrinsic structural imperfection. As an approximation, we take the same *S* for the above two scattering processes. We describe the detailed defination and calculation of *S* and *α*_QW_ in SI S2–S3. The last numerator and denominator represent the photon numbers absorbed by the QW for TM and TE polarized light, respectively.

### Photo-responsivity spectrum of the PCQWID and analysis

**Figure 2:**
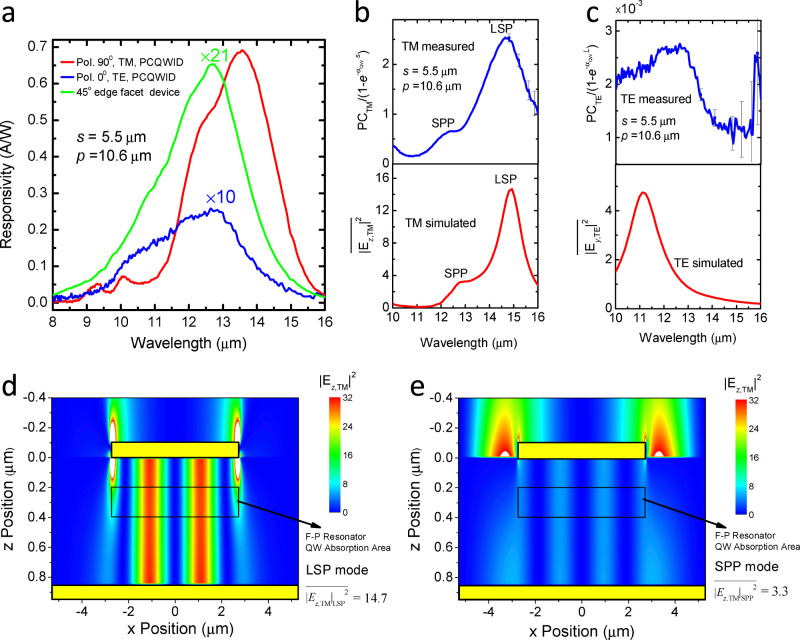
Photo responding properties and cavity mode behaviors of PCQWID. (a) Typical photocurrent spectra for TM (red) and TE (blue) light, together with that of 45 degree edge facet device (green). (b) Experimental (upper, blue) and simulated (lower, red) cavity mode dependence on wavelength for TM light. The experimental curve is the photo-responsivity spectrum divided by the quantum well absorption curve (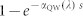). (c) Similar curves as (b) for TE light. The error bars show the large noise at wavelength larger than 15 μm in experiment. (d) Calculated intensity distribution of the square of electric field component in *z* direction of LSP cavity mode for TM light, |*E*_z,TM_|^2^, within the cavity. (e) Similar distribution as (d) for SPP cavity mode.

We investigate the polarization discriminating ability by illuminating the device with polarized IR light (with a polarizer whose extinction ratio is over 600 in the studied waveband) in Fourier transform IR spectrometer and by constructing the polarization azimuth dependence of photocurrent. The typical photocurrent responsivity spectra for TM and TE light are shown in Figure 2a in red and blue lines. Also shown is that of a 45 degree edge facet light-coupling device made from the same QW wafer as the reference in green line, which is the intrinsic QW absorption curve^14^. Photocurrent generation in PCQWID involves three processes including the light coupling into cavity structure and forming cavity modes, the scatterings of TM loss and TE leakage, and the photo-electric conversion in the QW. To understand the optical behaviors in the coupling cavity from photo-responsivity spectra, we need to extract the cavity characteristics by eliminating the absorption peak of the QW. For TM and TE light, the QW absorption curves in this cavity structure can be expressed as 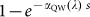 and 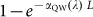, respectively, where *α*_QW_(*λ*) can be calculated from the photo-responsivity spectrum (green curve in Figure 2a) and the blackbody response of the 45 degree facet device. The detailed calculations can also be found in SI S4. We divide the PCQWID photo-responsivity curves with the respective absorptions, yielding the measured TM and TE mode spectra, as presented in the upper panels of Figure 2b and 2c. The spectrum of TM light demonstrates an obvious cavity mode behavior. There are two peaks at about 12.4 and 14.7 μm, which are attributed to SPP and LSP cavity modes^16^, respectively, in good agreement with the theoretical simulation of electric field intensity 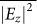 in the lower panel of Figure 2b that shows the SPP and LSP peaks at 12.8 and 14.9 μm. We will discuss these two cavity modes in the later text in details. As for TE light shown in the upper panel of Figure 2c, the very weak response (three orders of magnitude smaller than TM, near extinction condition in experiment) causes large noises, especially in the wavelength range larger than 15 μm, as indicated by the error bars. The overall trend shows a peak-like shape, similar with the calculated 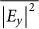 as shown in the lower panel of Figure 2c, although the peak position shifts by 1 μm.

Using Finite Difference Time Domain (FDTD) method and taking the input electric field intensity in the incident optical beam as the unity, the calculated distribution of the square of relative electric field components of TM light, |*E*_z,TM_|^2^, are shown in Figure 2d and 2e for both LSP and SPP modes. In which the average value of |*E*_z,TM_|^2^ is: 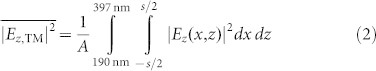
where *A* is the QW absorption area, *A* = 5.5 × 0.2 μm^2^ as indicated by the black boxes in Figure 2d and 2e. The calculated results show that 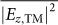 = 14.7 in LSP mode. The ratio of 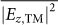 between LSP and SPP is 14.7/3.3 = 4.5, consistent with the experimental intensity ratio value of 4 that can be obtained from Figure 2b. As one can also see that in LSP mode the electrical field is localized under the metal strip to form Fabry-Perot resonances except those strongest parts condensed at the edges (Figure 2d) while in SPP mode it is weak and dispersive within the cavity with its strongest parts at the surface of the dielectric media (Figure 2e). The strong localization effect in LSP mode makes much higher |*E*_z,TM_|^2^, whose highest value can over 30, about 6 times higher than the maximum value of 5 in SPP mode inside the cavity. Similarly, the average relative values of electric field component for TE light, 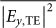, can also be calculated, which is 0.3 and 1.0 at the calculated TM LSP and SPP mode peak wavelengths of 14.9 and 12.8 μm, respectively. It is interesting to see that at the wavelength of TM LSP mode the average relative intensity of TM light is much higher than that of TE light (14.7/0.3), while at the wavelength of TM SPP mode the intensity ratio is only 3.3/1.0. According to Equation 1, the higher TM/TE ratio will yield higher extinction. This is one of the reasons we can obtain high extinction ratio in such a plasmonic micro-cavity structure, especially at the peak wavelength of LSP mode.

### Experimental polarization characteristics of PCQWID

**Figure 3:**
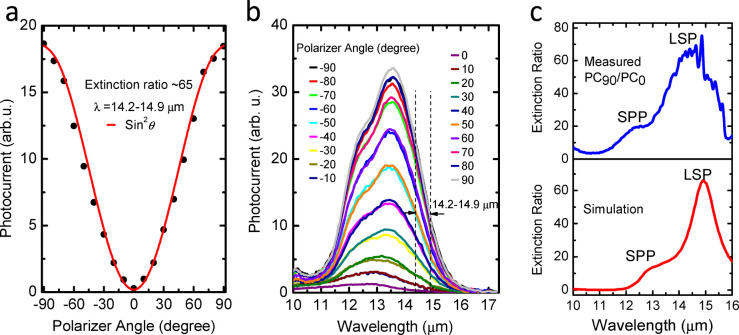
Polarization characteristics and extinction ratio of PCQWID. (a) Measured photocurrent average intensity at *λ* = 14.2~14.9 μm against incident light polarization angle. The red line shows normalized calculation of the square sine curve, showing a typical polarizer behavior. (b) the original photocurrent spectra vs. wavelength at different polarization angles. The photocurrent peak does not corresponding to the highest extinction ratio, as explained in the context. (c) Measured and simulated extinction ratio spectra for the PCQWID, indicating the maximum extinction ratio occurs at around the peak wavelength of LSP mode.

The experimental polarization characteristics of our PCQWID are shown in Figure 3a in black solid dots. The data are taken from the average photocurrent values at the wavelength range between 14.2–14.9 μm as indicated by the black dashed lines in Figure 3b, owing to the large fluctuations in the extinction ratio values (refer to Figure 3c for the measured extinction ratio values). Figure 3b is the photocurrent curves changing with the azimuth positions of the polarizer. The largest extinction ratio does not occur at the peak wavelength of 13.5 μm, at which the photocurrent is the largest. This is because that the photocurrent curves are modulated by both cavity modes and the QW absorption peak. In our experiment the QW absorption peak is at about 12.5 μm (green line in Figure 2a) and the cavity mode peak is at 14.7 μm. The averaged experiment values in solid dots agree with the normalized square of sine function curve in red line, which is a standard characteristics of a polarizer, indicating a perfect polarization discriminating behavior of the device. Figure 3c is the measured and simulated extinction ratio dependence on the wavelength. The measured curve in blue line is obtained by dividing the two photocurrent response spectra at polarizer angles of 90 and 0 degrees in Figure 3b. The maximum extinction ratio appears at the wavelength around 14.7 μm, close to the simulated TM LSP peak wavelength. The simulated curve is from the calculation of the coupled TM and TE optical power inside the cavity and their absorptions (see SI S5). It agrees well with the experiments, reproducing the two peaks of LSP and SPP at the respective wavelength and their relative values, verifying the analysis that we proposed for describing the polarization extinction ratio dependence in PCQWID, which provides guidance for further optimization of the extinction ratio.

The above cavity and polarization results quantitatively demonstrate the cavity-mode effects in PCQWID and strongly suggest that for a high extinction ratio one should choose the PCQWID responding peak wavelength to be at that of the LSP mode. Moving the TE peak to shorter wavelength by decreasing the grating period while retaining the metal strip width will decrease the TE intensity at the LSP wavelength and will further increase the extinction ratio. In addition, tuning the QW absorption peak to the LSP resonant wavelength by adjusting the intersubband transition energy is also important so as to increase the QW absorption, according to Equation 1.

### Cavity mode of LSP and SPP

**Figure 4:**
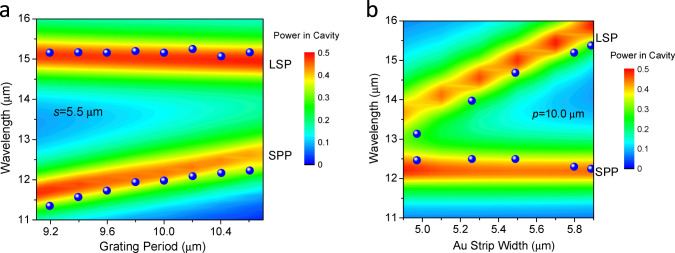
Cavity mode dependence on the grating parameters of PCQWID. Blue spheres are experimental data taken from the peaks in LSP and SPP mode spectra, as the one shown in the upper panel of Figure 2b. (a) With different periods at Au strip width *s* = 5.5 μm. (b) With different Au strip widths at period *p* = 10.0 μm.

To clarify the cavity mode behavior of LSP and SPP, we have studied the mode variations against the grating parameters. LSP resonance is resulted from local electron density oscillation around a single metal strip or sphere^17^, and is strongly dependent on metal size^16^. It couples the incident light from free space, transferring the light energy into the cavity, supporting the propagating TM wave to form Fabry-Perot resonantors^18^. This kind of surface plasmons, similar with those in metal holes and spherical particles^16^, does not require the wave vector matched excitation and is independent to periodicities. SPP resonance is propagating EM waves coupled to the electron plasma of a conductor at a dielectric interface^18^. In our one-dimensional grating structure, the periodic array provides the necessary plane wavevector to excite SPP mode. From FDTD analysis, the LSP resonant wavelength increases with the increasing width *s* and almost unchanged with period *p*. While the SPP resonant wavelength is almost independent on *s* and increases with the increasing *p.* These properties have been reproduced in series of PCQWID devices as shown in Figure 4a and 4b, where the blue spheres are the experimental peak wavelengths of LSP and SPP modes taken from the mode spectra (One of the measured mode spectra for *p* = 10.6 μm and *s* = 5.5 μm is shown in the upper panel of Figure 2(b). The colored maps are the calculations of the optical power in the cavity, which indicates obvious concentrations at the resonant wavelengths of LSP and SPP. The experimental data are almost overlapped over the calculated peaks, confirming our attributions of the LSP and SPP modes in the plasmonic micro-cavity. Therefore it is clear that we have observed the co-existence of LSP and SPP in experiment. The reason would be attributed to the relatively thick of the cavity thickness. For our PCQWID rather than plasmonic micro-cavity absorbers^19^ such a co-existence is very important for sustaining quantum efficiency of the detectors, here we call it a two-mode manipulation, which enables the simultaneous tailoring on the two modes by adjusting the parameters of the cavity. LSP resonance yields high extinction ratio, the importance of SPP mode, however, should not be neglected. Comparing with the ultra-thin absorbers^19^, the cavity thickness of our device cannot be reduced to several nanometers so as to contain the photo-electric conversion active layers and allow the wave propagation to retain quantum efficiency of photo-electric conversion. The enhancement factor can be as high as 1.25 (see SI S4.3). Also notice that the QW absorption is weak because the increment of QW absorption will need to increase the doping level in the well and will cause heightening of the dark current. The assistance of SPP mode helps to keep good quantum efficiency in photoelectric conversion, making it to be ~6% with only single QW, similar with that of ~5% in conventional 50 periods QWIPs.

## Discussion

We have demonstrated the high infrared light polarization discriminating capabilities of metal-QW-metal plasmonic micro-cavities. The photocurrent response spectra directly and effectively disclose the behavior of the optical waves inside the cavity. Benefited from double-selection-rules of the plasmonic cavity and the QW, we observe well-enhanced polarization extinction ratio of about 65 at LWIR of 14.7 μm. The maximum extinction ratio appears at the wavelength close to the LSP resonant mode, in good agreement with the theoretical calculations. Future studies will include tailoring the micro-cavity to enable circular polarization discriminating by chiral structures. It is reasonable to expect that our PCQWID structure can be employed for FPA device, in which the constraint of cavity resonance would reduce the side-edged light leakage and maintain high pixel-level extinction ratio, to achieve high quality polarimetric image in LWIR range.

## Method

### Fabrication of plasmonic micro-cavity quantum well infrared detector (PCQWID)

The fabrication starts on GaAs/AlGaAs single quantum well (QW) epitaxial layers substrated on a GaAs wafer. It was defined into 250 × 350 μm^2^ mesas by standard photolithography and chemical etching. The mesa depth was controlled to reach the bottom contact layer. A GeAu(100 nm)/Ni(20 nm)/Au(400 nm) layer was deposited by electron-beam evaporation and patterned on the mesas as the top electrode and on the side bottom contact layer as the bottom common electrode. Then a rapid thermal annealing was processed to form Ohmic contact. Another Ti(20 nm)/Au(80 nm) layer was deposited and lift-offed to form the surface plasmon grating. The patterned surface was then bonded on a GaAs polishing wafer. Following that, a series of mechanism polishing and selective wet etching using the mixture of citric acid (C_6_H_8_O_7_) and hydrogen peroxide (H_2_O_2_) were performed to remove the 600 μm thick GaAs substrate and reaching the Al_0.55_Ga_0.45_As etch stop layer. Then a reflection Ti(20 nm)/Au(80 nm) layer was deposited as the bottom reflection layer of the cavity. This bottom side was fixed on a sapphire wafer with UV curing adhesive. The preparation finished with a de-bonding process from the GaAs polishing wafer. The flowchart for preparing the PCQWID is shown in Figure S1.

## Author Contributions

Q.L. performed FDTD calculation and designed and fabricated the plasmonic cavity quantum well infrared detector prototypes and conducted all the measurements. Z.F.L. and N.L. supervised the fabrications and measurements. X.S.C. assisted in the design and numerical modelling of PCQWID. P.P.C. grew the quantum well epitaxy layers. W.L. proposed the physical idea and organized the research. X.C.S. supervised the project. All authors discussed the results and commented on the manuscript.

## Supplementary Material

Supplementary Information Supplementary Information
