# Supplementary material for: High-Polarization-Discriminating Infrared Detection Using a Single Quantum Well Sandwiched in Plasmonic Micro-Cavity
Source: Sci Rep. 2014 Sep 11;4:6332. doi: 10.1038/srep06332 (PMC4160703; doi:10.1038/srep06332)
Supplement: Supplementary Information [file srep06332-s1.pdf]

Supplementary Information

High-polarization-discriminating infrared detection using a single quantum well sandwiched in plasmonic micro-cavity

Qian Li, ZhiFeng Li, Ning Li, XiaoShuang Chen, PingPing Chen, XueChu Shen and Wei Lu*

National Laboratory for Infrared Physics, Shanghai Institute of Technical Physics, Chinese Academy of Sciences, 500 Yutian Road, Shanghai 200083, P. R. China and Synergetic Innovation Center of Quantum Information & Quantum Physics, University of Science and Technology of China, Hefei, Anhui 230026, P. R. China

*E-mail: luwei@mail.sitp.ac.cn

1. Fabrication of plasmonic micro-cavity quantum well infrared detector (PCQWID)

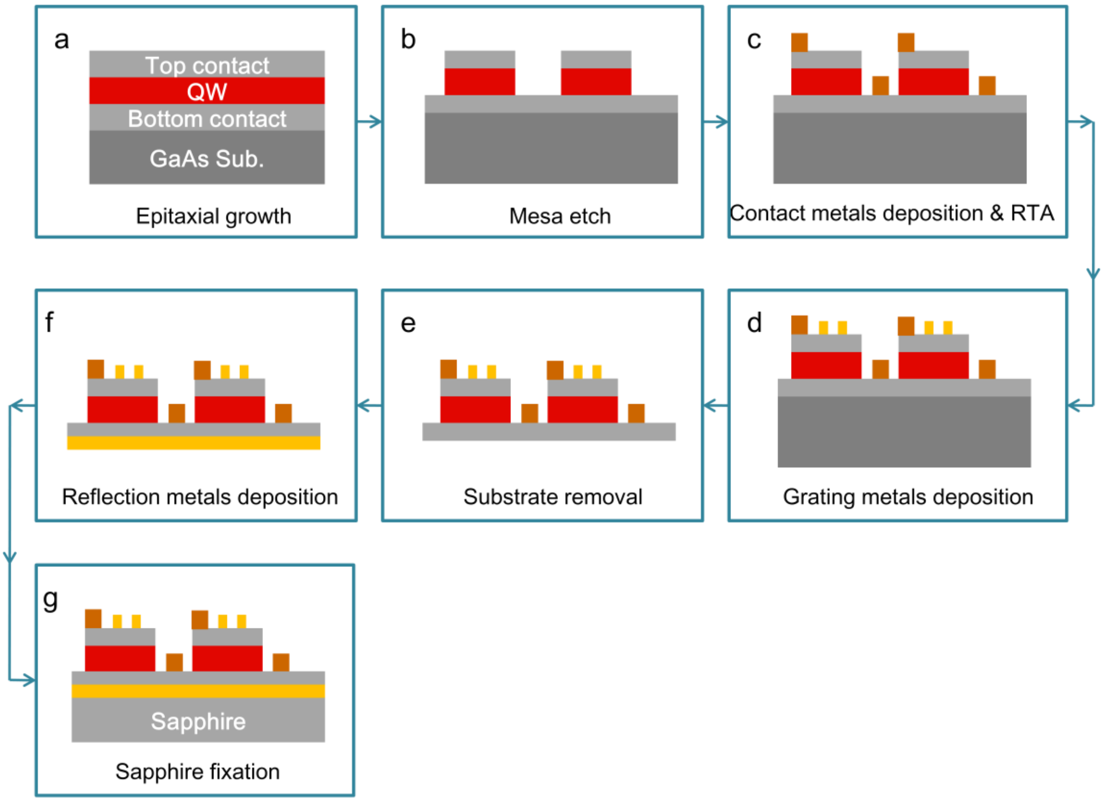

Figure S1 | Schematic flowchart for the fabrication procedure of PCQWID.

2. QW absorption coefficient

QW absorption coefficient $\alpha_{\mathrm{QW}}(\lambda)$ can be obtained from its photocurrent responsivity spectrum (shown in Figure S2, calculated by measuring its blackbody response and photocurrent spectrum) of the 45 degree edge facet coupling device made from the same expitaxial wafer as for PCQWID. With equation $R(\lambda) = \frac{q}{h\nu}\eta(\lambda) = \frac{q\lambda}{hc}\eta(\lambda)$[1], where q is elementary charge, c is velocity of light in vacuum, h is Plank constant, quantum efficiency $\eta(\lambda)$ can be obtained. With another equation $\eta(\lambda) = \frac{1-e^{-2\alpha_{\mathrm{QW}}(\lambda) l_{45^0}}}{2}$[1], where $l_{45^0} = 207\ \mathrm{nm}$ is the thickness of QW (To compare the absorption coefficient value with Ref. [1] we use the same absorption model as two barrier 100 nm and one well 7 nm), we can get the QW absorption coefficient $\alpha_{\mathrm{QW}}(\lambda)$.

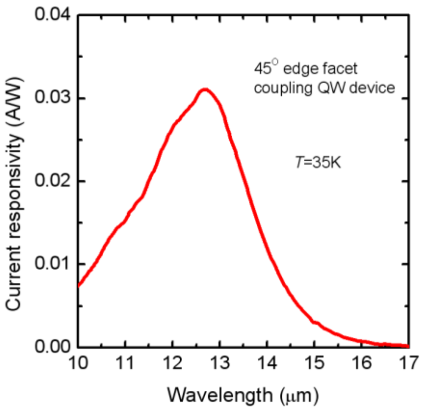

Figure S2 | Current responsivity spectrum of the 45 degree edge facet light-coupling QW detector.

3. Definition and calculation of the scattering factor *S*

In the article we define the value of scattering factor S to represent the ratio of E_z scattering into E_y in TM polarized light (TM loss). It can be writen as $S = \frac{\overline{|E_{y,S,\mathrm{TM}}(\lambda)|^2}}{\overline{|E_{z,\mathrm{TM}}(\lambda)|^2}}$. The subscript S is to indicate that this part of E_y is from the scattering of E_z. Similarly, for E_y scattering into E_z in TE polarized light (TE leakage) we

have $S = \frac{\overline{|E_{z,S,\mathrm{TE}}(\lambda)|^2}}{|E_{y,\mathrm{TE}}(\lambda)|^2}$. The scatterings are resulted from the extrinsic structural imperfection, such as the finite length of the grating (side boundary) and the unsmooth in the grating metal strips, and should be regarded as a small perturbation. As an approximation we arbitrarily take the same S for TM and TE light. S is calculated by taking the measured extinction ratio at the Fabry-Perot resonant wavelength (*i.e.*, LSP mode wavelength λ=14.9 μm) into Eq. (1). With the enhancement factor G being calculated later in this text, all the other values in Eq. (1) can be obtained, then we get S=0.004 (at λ=14.9 μm). Its small value agrees the perturbation assumption.

4. The QW absorption model within the Fabry-Perot resonator and the SPP mode assisted enhancement

4.1 The QW absorption within the Fabry-Perot resonator

Because of the strong impedance mismatch between the single-metal and double-metal regions, a lateral Fabry-Perot resonator has been formed under the metal strip[2]. The coupled electromagnetic (EM) waves will be trapped in the resonator and be multiply reflected between the two interfaces, as shown in Fig. S3, with the resonant frequency $\nu_K = \frac{cK}{2n_M s}$ [2]. This frequency turns out to be consistent with the resonant frequency for LSP mode in our device, confirming the LSP coupling mechanism. The effective refraction index n_M at the resonant frequency can be calculated to be $n_M = 4.1$. The EM waves will be absorbed in the paths of the each reflection, as depicted in Fig. S3.

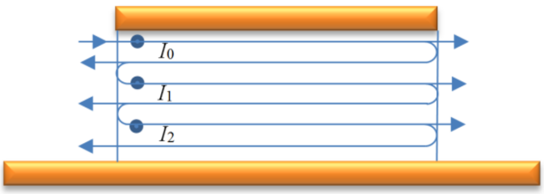

Figure S3 | Schematic diagram for optical beam multiply reflected and absorbed by QW within the Fabry-Perot resonator.

For TM light illumination the photo-current responsivity R_x is proportional to the absorbed optical power ΔI_{TM}. We can calculate the total absorption by analyzing the absorption at each path. Taking the reflectivity at the F-P resonator interface as R, at the resonant wavelength, the optical absorption can be written as:

$$\Delta I_1 = I_0 - I_1 = I_0\,(1 - e^{-\propto_{\mathrm{QW}} s})(1 + Re^{-\propto_{\mathrm{QW}} s}),$$

$$\Delta I_2 = I_1 - I_2 = I_0 R^2 e^{-2\propto_{\mathrm{QW}} s}\,(1 - e^{-\propto_{\mathrm{QW}} s})(1 + Re^{-\propto_{\mathrm{QW}} s}),$$

$$\cdots$$

$$\Delta I_n = I_{n-1} - I_n = I_0 (R^2 e^{-2\propto_{\mathrm{QW}} s})^{n-1}\,(1 - e^{-\propto_{\mathrm{QW}} s})(1 + Re^{-\propto_{\mathrm{QW}} s}).$$

The total absorption power for the TM illumination within the F-P resonator is:

$$\Delta I_{\mathrm{TM}} = \sum_{n=1}^{\infty} \Delta I_n = I_0 \frac{1 - e^{-\propto_{\mathrm{QW}} s}}{1 - Re^{-\propto_{\mathrm{QW}} s}}.$$

For the F-P resonator, the maximum average effective optical power $I_{\max} = 0.35 \times I_i \overline{\left|E_{z,\mathrm{TM}}\right|^2}$, where 0.35 is the transmissivity of the polarizer used in the experiment, I_i is the total incident power. $\overline{\left|E_{z,\mathrm{TM}}\right|^2}$ is the relative average E_z power whose value is calculated as taking the input electric field intensity in the incident optical beam as the unity. As $I_{\max} = \frac{I_0}{(1-R)^2}$ [3], we have

$$I_0 = I_{\max}(1-R)^2 = 0.35 \times \overline{\left|E_{z,\mathrm{TM}}\right|^2} I_i (1-R)^2,$$

$$\Delta I_{\mathrm{TM}} = \frac{0.35 \times \overline{\left|E_{z,\mathrm{TM}}\right|^2}\, I_i (1-R)^2 (1 - e^{-\propto_{\mathrm{QW}} s})}{1 - Re^{-\propto_{\mathrm{QW}} s}}.$$

Because of $\propto_{\mathrm{QW}} s \ll 1$, $1 - Re^{-\propto_{\mathrm{QW}} s} \approx 1 - R$, and we obtain the total absorption power for the TM light:

$$\Delta I_{\mathrm{TM}} = 0.35 \times \overline{\left|E_{z,\mathrm{TM}}\right|^2}\, I_i (1-R)(1 - e^{-\propto_{\mathrm{QW}} s}).$$

4.2 Reflectivity *R*

The finesse of an F-P resonator is defined as $f = \frac{\pi R^{\frac{1}{2}}}{1-R}$ [3], where R is the

reflectivity at the F-P resonator interfaces. So we have $R=\frac{2+(\frac{\pi}{f})^2-\sqrt{(2+\frac{\pi^2}{f^2})^2-4}}{2}$. We also have f= $\frac{\nu_f}{\Delta\nu}$, where $\nu_f=\frac{c}{2n_{\mathrm{M}}d}$ is the free spectral range of the cavity, defined as the frequency spacing between two successive reflected optical intensity minima. n_{M} is the effective refractio index. d is the cavity length. From the FDTD simulated reflection spectrum, as shown in Fig. S4, we have the averaged $\nu_f=\frac{1}{2}\times\frac{445+376}{2}=205\ \mathrm{cm}^{-1}$. $\Delta\nu$ is the full width at half maximum of the resonant peak and is simulated to be 55 cm^{-1}. Therefore we have $f=\frac{\nu_f}{\Delta\nu}=3.7$. Then R=0.45.

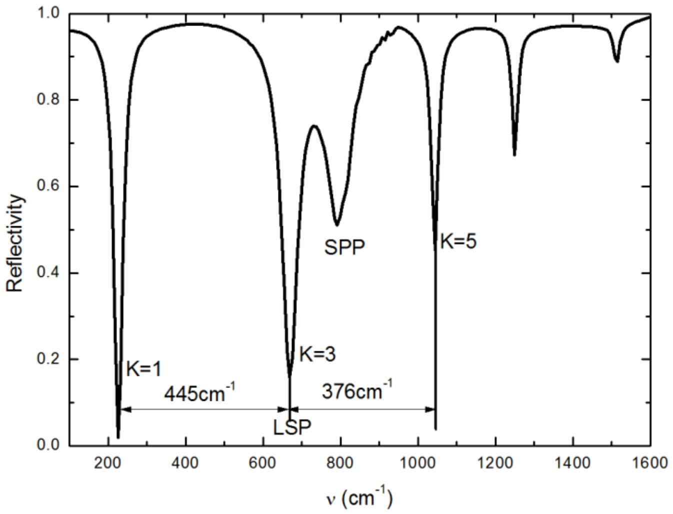

Figure S4 | FDTD calculated reflection spectrum of the plasmonic cavity.

4.3 Modification of the QW absorption within the Fabry-Perot resonator and the enhancement factor *G*: the SPP assisted enhancement

For the 45 degree edge facet coupled QW device, which is taken as a standard reference in studying the new coupling scheme, the surface reflectivity is $R_{45^o}=(\frac{1-n_{\mathrm{GaAs}}}{1+n_{\mathrm{GaAs}}})^2=29\%$. Its absorbed optical power ΔI_{45^o} is:

$$\Delta I_{45^o}\approx(1-R_{45^o})\,I_i\,(1-e^{-\propto_{\mathrm{QW}}d_{45^o}})$$

where d_{45^o} is the effective absorption length, $d_{45^o}=2\times207$ nm.

At the QW peak absorption wavelength of 14.7 μm, $\propto_{\mathrm{QW}}=30\ \mathrm{cm}^{-1}$, $1-e^{-\propto_{\mathrm{QW}}s}\approx\propto_{\mathrm{QW}}s$, $1-e^{-\propto_{\mathrm{QW}}d_{45^o}}\approx\propto_{\mathrm{QW}}d_{45^o}$. Taking the previous expression

for ΔI_{TM} we have the ratio of the absorbed optical power between the TM light illuminated plasmonic cavity device and the 45^0 edge facet coupled device:

$$\frac{\Delta I_{\text{TM}}}{\Delta I_{45^0}} = \frac{0.35\times\overline{|E_{z,\text{TM}}|^2}\, I_i\,(1-R)(1-e^{-\propto_{\text{QW}} s})}{0.71\; I_i\,(1-e^{-\propto_{\text{QW}} d_{45^o}})} = \frac{0.35\times 14.7\, I_i\,(1-R)\propto_{\text{QW}} s}{0.71\, I_i \propto_{\text{QW}} d_{45^o}} = 53.$$

Because $R_{\text{TM}} \propto \Delta I_{\text{TM}}$, $R_{45^0} \propto \Delta I_{45^0}$, we have $\frac{R_{\text{TM}}}{R_{45^0}}(\text{theory}) = \frac{\Delta I_{\text{TM}}}{\Delta I_{45^0}} = 53.$

In experiment, however, the observed ratio is $\frac{R_{\text{TM}}}{R_{45^0}}(\text{experiment}) = 66.5$, which is 1.25 times the theoretical value. This is attributed to that in each reflection at the interface of F-P resonator there will be some transmissions to exist, as indicated by the reflectivity of R=0.45. These multiply leaked transmissions will be spread, assisted by the SPP mode, across the lateral F-P resonator and get into the neighboring regions. This part of EM waves will also be absorbed by the QW and contribute to the photocurrent. Therefore we introduce an enhancement factor G in the QW absorption model to express the SPP assisted propagation enhancement:

$$G = \frac{R_{\text{TM}}(\text{experiment})}{R_{\text{TM}}(\text{theory})} = \frac{\frac{R_{\text{TM}}}{R_{45^0}}(\text{experiment})}{\frac{R_{\text{TM}}}{R_{45^0}}(\text{theory})} = \frac{66.5}{47} = 1.25.$$

The modified TM absorption will be written as

$$\Delta I_{\text{TM}} = 0.35 \times \overline{|E_{z,\text{TM}}|^2} I_i(1-R)(1-e^{-\propto_{\text{QW}} s})G.$$

5. Physical meaning of Equation (1) and the calculation of extinction ratio dependence on wavelength (extinction ratio line shape)

5.1 Extinction ratio at the resonant wavelength and the physical meaning of Equation (1)

To calculate the extinction ratio in our PCQWID at the LSP mode resonant peak wavelength in the Fabry-Perot resonator we propose Equation (1) as

$$\rho = \frac{R_x}{R_y} = \frac{P_{0,\text{TM}}\,(1-R)(1-\mathrm{e}^{-\propto_{\text{QW}} s})\cdot G}{P_{0,\text{TE}}\,(1-\mathrm{e}^{-\propto_{\text{QW}} L})} = \frac{0.35\times\overline{|E_{z,\text{TM}}|^2}\,(1-S)\,(1-R)(1-\mathrm{e}^{-\propto_{\text{QW}} s})\cdot G}{\overline{|E_{y,\text{TE}}|^2}\,S\,(1-\mathrm{e}^{-\propto_{\text{QW}} L})} \quad (1)$$

where the extinction ratio is defined as the ratio of photo-responses, R_x/R_y, along the two orthogonal TM and TE polarization directions. $P_{0,\text{TM}}$ and $P_{0,\text{TE}}$ are the effective E_z in TM and TE light, respectively. G and S are enhancement and scattering factors.

R is the lateral reflectivity at the interface between the single-metal and double-metal regions. s and L are the widths of metal strip and detector mesa, respectively. $\overline{|\mathrm{E}_{z,\mathrm{TM}}|^2}$ and $\overline{|\mathrm{E}_{y,\mathrm{TE}}|^2}$ are the simulated average relative intensities of electric field by taking the input electric field intensity in the incident optical beam as the unity, respectively. From previous discussion of the modified F-P resonator absorption model, at the resonant wavelength the photo-response under TM light illumination, R_x, can be written as $R_x = 0.35 \times \overline{|E_{z,\mathrm{TM}}|^2}(1-R)(1-e^{-\propto_{\mathrm{QW}} s})G$. Taking into account of the effect of the scattering factor S, we have $R_x = 0.35 \times \overline{|E_{z,\mathrm{TM}}|^2}(1-S)(1-R)(1-e^{-\propto_{\mathrm{QW}} s}) \cdot G$. Similarly, under TE light illumination, the photoresponse comes from the photo-absorption in QW, and is proportional to TE leakage phonton numbers, and therefore proportional to the effective optical power $P_{0,\mathrm{TE}}$, which is the average power of E_z component within the QW absorption area. The effective power of TE leakage is $P_{0,\mathrm{TE}} = \overline{|E_{y,\mathrm{TE}}|^2} \cdot S$ and its QW absorption is $(1-e^{-\propto_{\mathrm{QW}} L})$. The scattering factor S is small, the most part of un-scattered TE light can propagate to the mesa boundary. So the absorption length for TE light can be taken as the mesa width L=230 μm. Therefore we can write the extinction ratio equation as

$$\rho = \frac{R_x}{R_y} = \frac{0.35 \times \overline{|E_{z,\mathrm{TM}}|^2}\,(1-S)\,(1-R)(1-\mathrm{e}^{-\propto_{\mathrm{QW}} s}) \cdot G}{\overline{|E_{y,\mathrm{TE}}|^2}\,S\,(1-\mathrm{e}^{-\propto_{\mathrm{QW}} L})}.$$

We do not consider the quantum efficiency in photoelectric conversion process because it is the same for both TM and TE light and will be canceled in the equation.

5.2 Extinction ratio line shape calculation

As QW can only absorb E_z component, when taking into account of the scattering factor, we only consider the contribution of E_z. At any wavelength, the coupled optical power inside the PCQWID for TM light is proportional to $\overline{|E_{z,\mathrm{TM}}(\lambda)|^2}\,(1-S)$. The optical absorption is proportional to $(1-e^{-\propto_{\mathrm{QW}}(\lambda) s})$. For TE light, its coupled optical power is $\overline{|E_{y,\mathrm{TE}}(\lambda)|^2} S$, with the optical absorption in

proportion to $(1-e^{-\propto_{\mathrm{QW}}(\lambda)L})$. Therefore the extinction ratio $\rho(\lambda) \propto \frac{\overline{|E_{z,\mathrm{TM}}(\lambda)|^2}\,(1-S)\,(1-e^{-\propto_{\mathrm{QW}}(\lambda)s})}{\overline{|E_{y,\mathrm{TE}}(\lambda)|^2}\,S(1-e^{-\propto_{\mathrm{QW}}(\lambda)L})}$. Assuming the scattering factor S is independent to the wavelength, the line shape of the extinction ratio against wavelength should be approximately the same as $\frac{\overline{|E_{z,\mathrm{TM}}(\lambda)|^2}\,(1-e^{-\propto_{\mathrm{QW}}(\lambda)s})}{\overline{|E_{y,\mathrm{TE}}(\lambda)|^2}\,(1-e^{-\propto_{\mathrm{QW}}(\lambda)L})}$. Taking the experimental maximum value of 65 at 14.9 μm as the simulation maximum, we can obtain the simulated extinction ratio change against wavelength as indicated in Fig. 3(b), which is well agreed with the experiments.

References

1. Levine, B. F., Quantum-well infrared photodetectors, *J. Appl. Phys*, **74**(8), R1-R81 (1993)
2. Todorov, Y. *et al*. Strong light-matter coupling in subwavelength metal-dielectric microcavities at terahertz frequencies. *Phys. Rev. Lett*. **102**, 186402 (2009).
3. Saleh, B. E. A., Teich, M. C. *Fundamentals of Photonics*, (Wiley, 1991, pp. 317)
